# Spray-Dried Plasma Promotes Broiler Chick Growth by Enhancing Immune Surveillance

**DOI:** 10.3390/ani13091436

**Published:** 2023-04-22

**Authors:** Candice E. C. Blue, Yasin Jababu, Salam A. Ibrahim, Radiah C. Minor, Leonard L. Williams, Adedeji O. Adetunji, Rizwana Ali, Lea S. Young, Yewande O. Fasina

**Affiliations:** 1 Department of Animal Sciences, North Carolina Agricultural and Technical State University, Greensboro, NC 27411, USArcminor@ncat.edu (R.C.M.); aoadetunji@ncat.edu (A.O.A.);; 2 Food Microbiology and Biotechnology Laboratory, Food and Nutritional Science Program, North Carolina Agricultural and Technical State University, Greensboro, NC 27411, USA; ibrah001@ncat.edu; 3 Center for Excellence in Post-Harvest Technologies, North Carolina Research Campus, Kannapolis, NC 28081, USA; llw@ncat.edu; 4 Prestage Department of Poultry Science, North Carolina State University, Raleigh, NC 27695, USA; rizwanaali2009@gmail.com

**Keywords:** spray-dried plasma, growth performance, immunocompetence, bifidobacteria, immunoglobulin, heterophils, broiler chicken

## Abstract

**Simple Summary:**

Over the years, the poultry industry has relied on the use of in-feed antibiotics as a growth-promoting agent and for the prevention of diseases. However, antibiotic use has brought about pathogens that are resistant to antimicrobials. To this end, spray-dried plasma (SDP), an animal blood by-product that is rich in protein-containing lipids, peptides, immunoglobulins, transferrin, and fibrinogen, is being explored as a replacement for in-feed antibiotics in poultry. We evaluated the immunological and biochemical profile of SDP in order to understand how it enhanced performance values when supplemented to a broiler diet. At the end of the four-week study, our findings demonstrated a decrease in the number of heterophils and an increase in immunoglobulin in circulation, with oxidative stress falling in the normal range. Bifidobacteria counts also increased in the SDP-supplemented treatment. This demonstrated that SDP supplementation prevented infection and caused an increase in immunoglobulin concentration required to support intestinal development and gut microbiota modulation.

**Abstract:**

Spray-dried plasma (SDP) contain a variety of functional proteins that play an immunomodulatory role. To evaluate the potential of SDP to stimulate the immune system, day-old Ross 708 male broiler chicks (200) were allocated randomly to five dietary treatments. Treatment 1 (CX) comprised chicks fed basal unmedicated corn–soybean meal (SBM) without the addition of SDP. Treatment 2 (MX) includes chicks fed unmedicated corn–SBM basal containing Bacitracin methylene disalicylate (BMD) at 0.055 g/kg diet. Treatments 3 (SDP1), 4 (SDP2), and 5 (SDP3) contained chicks given unmedicated corn–SBM basal, into which SDP was included at 10, 20, and 30 g/kg diet, respectively. On d 7, 14, and 21, chicks’ body weight and FCR were calculated. Additionally, leucocyte counts, oxidative status, and IgY concentrations were determined in blood. On d 23, fecal populations of selected indicator bacteria species were determined. Results showed that FCR for SP3 was superior (*p* < 0.05) to other treatments. Likewise, heterophil numbers decreased in MX and SDP treatments compared to CX. Circulating IgY concentration was higher for SDP dietary treatments (*p* < 0.05) compared to MX. In conclusion, dietary SDP at 30 g/kg enhanced immune surveillance by increasing circulating IgY levels, maintaining a normal oxidative state, and increasing gut Bifidobacteria, thereby improving chick growth performance.

## 1. Introduction

Increasing broiler chicken performance while ensuring the health and wellbeing of the birds has been the primary goal of the poultry industry. Over the years, the industry has relied on the use of in-feed antibiotics as a growth-promoting agent and for the prevention of diseases [1]. However, the use of antibiotics has led to the emergence of pathogens that are resistant to antimicrobials. In a bid to use safer alternatives to antibiotics in the improvement of growth performance and immune response, feed supplements and additives such as plant extracts and biogenics have been used [2,3].

Swine producers have consistently utilized spray-dried plasma (SDP) to enhance the growth performance, intestinal health, and survival of piglets [4]. Moreover, as a protein source in animal feed, SDP has been reported to promote livestock health [5]. Spray-dried plasma (SDP) is an animal blood by-product rich in protein and derived from the blood of healthy porcine or bovine animals through the separation of plasma from whole blood using the centrifugation method [6]. SDP contains active components such as amino acids, enzymes, lipids, peptides, immunoglobulins, transferrin, fibrinogen, and growth factors, which play a role in diverse biochemical and immunological processes [7]. It has also been suggested that the immunoglobulin-rich fraction in plasma may be responsible for the beneficial effects attributed to SDP [8]. Because the plasma is recovered from the blood that was collected from a healthy animal, the plasma is deemed safe. Moreover, the spray-drying process effectively destroys potential viral and bacterial pathogens that may be present [4]. To this end, the use of SDP in livestock feed is safe from a public health perspective [5].

Extensive studies focused on evaluating the immune-enhancing and gut microbiota regulatory status of SDP in rats, and pigs have provided evidence of its influence as a growth promoter and in the suppression of inflammation [9,10]. In addition, it has been reported that regulation of the immune system is one of the many roles of the gut microbiota [11]. In a previous study, we demonstrated that porcine SDP supplementation at 30 g/kg diet and BMD antibiotic (at 0.055 g/kg diet) had similar effects in reducing intestinal *Salmonella* spp. colonization in broiler chickens [12]. However, the role of dietary SDP supplementation in enhancing the immune response and regulating broiler chicken gut microbiota has not been explored. Therefore, the current study was conducted in order to determine the effects of SDP at graded levels on pro-oxidant capacity, gut microorganisms, and immune response in broiler chickens. These data will provide an understanding of the mechanism by which SDP enhances the animals’ growth performance and health.

## 2. Materials and Methods

The animal care and use procedures were approved by the Institutional Animal Care and Use Committee (IACUC) of North Carolina Agricultural and Technical State University.

### 2.1. Experimental Design, Diet, and Bird Management

In a 4-week experiment, day-old Ross 708 male broiler chicks (200) commercially sourced chicks were randomly allocated to five treatments in a completely randomized design (CRD). Treatment 1 (CX) consisted of chicks fed corn–soybean meal (SBM) basal without SDP. Treatment 2 (MX) consisted of chicks given corn–SBM basal into which Bacitracin methylene disalicylate (BMD; Zoetis Services LLC, Parsipanny, NJ, USA) was added at 0.055 g/kg diet. Treatments 3 (SDP1), 4 (SPD2), and 5 (SDP3) comprised chicks fed unmedicated corn–SBM basal into which SDP was included at 10, 20, and 30 g/kg diet, respectively (Table 1). Experimental diets were manufactured at the North Carolina State University Feed Mill (Raleigh, NC, USA) according to the feed formula (Table 1). Each treatment consisted of 4 replicate pens, with 10 chicks/pen fed ad libitum and allowed free access to water throughout the experiment. The pens contain a hanging feeder, a nipple drinker line, and fresh unused bedding. The SDP used in this study is a kind gift from APC Incorporated (Ankeny, IA, USA). In addition, the bird housing was set at a temperature of 92 °F from d 1 to d 7, and 87 °F from d 8 to d 21. Subsequently, it was reduced to 77 °F up to 28 d. Photoperiod consisted of continuous (23L: 1D) lighting at 30 lux from placement to 21 d; thereafter, it was reduced to 12L: 12D lighting up to 28 d. All experimental diets were formulated to meet or slightly exceed nutrient requirements based on the recommendations in the Ross broiler nutrition specification handbook [13]. The diets were fed as crumbles throughout the duration of the experiment.

### 2.2. Growth Performance

Bodyweight (BW), body weight gain (BWG), and feed intake (FI) of chicks were recorded on d 7, d 14, and d 21, and the feed conversion ratio (FCR) was calculated.

### 2.3. Blood and Plasma Collection and Preparation

On d 14 and d 25 of experiment, two birds were randomly taken from each pen, and blood was collected from the brachial (wing) vein using a sterile 23 gauge 1″ needle attached to pre-labeled sterile EDTA vacutainer tubes. The blood samples were then centrifuged at 1500× *g* for 10 min., and the plasma (supernatant) was collected and stored at −80 °C.

### 2.4. Differential Leukocyte Count Analysis

On d 14, a thin smear of each blood sample was created on glass slides that were subsequently stained using the HEMA 3 Wright-Giemsa staining kit (Fisher Scientific, Waltham, MA, USA) based on manufacturer’s instructions. The smear on each slide was allowed to dry, and then a drop of immersion oil was added onto the slide and viewed under the microscope (100× magnification, DME Side by Side Pathology 2×,). (Leica microsystems Inc., North Deerfield, IL, USA). Leukocytes (100 per slide) were counted, and the percentage of heterophil and lymphocyte was calculated in addition to the heterophil: lymphocyte ratio as previously described by [14].

### 2.5. Evaluation of Phaseolus Vulgaris-P-Induced Cutaneous Delayed-Type Hypersensitivity (DTH)

On d 25, the DTH analysis was performed on 2 birds/replicate pen, totaling 8 birds per treatment. With the aid of a constant tension micrometer caliper, the thickness of the toe web between the second and third digits of both feet was measured on d 24 before *Phaseolus vulgaris* (red kidney bean, PHAP) was injected. This data gave the initial pre-injection reading. Thereafter, PHAP (100 µL of 1 mg/mL; Sigma-Aldrich Inc., St. Louis, MO, USA) was injected into the right foot of the bird between the second and third digits. The left foot served as control and was injected with 100 µL of sterile PBS. At 24 h post-injection, the thickness of the toe web was measured with the aid of a micrometer.

### 2.6. Assay of Total IgY Concentration

Blood plasma was assayed for total IgY concentration. Plasma IgY concentrations were determined with the aid of a commercial sandwich ELISA kit (450 nm; E33-104, Bethyl Laboratory, Montgomery, TX, USA) based on manufacturer’s instructions on d 25. The immunoglobulin concentrations were determined relative to standard curve and expressed in nanograms per milliliter (ng/mL).

### 2.7. Pro-Oxidant Capacity

On d 14, a reactive oxygen metabolites (d-ROMs) test (Diacron International s.r.l., Grosseto, Italy) measured using the FREE DUO system (Diacron International s.r.l., Grosseto, Italy) was employed to assess the pro-oxidant capacity of the plasma samples for each treatment. Based on the existing reference level, a pro-oxidant capacity >27.20 mg H_2_O_2_/dL was considered a high level of oxidative stress, as described by [15].

### 2.8. Microbiological Analysis of Fecal Microbiota

On d 23, 10 g of fecal samples collected by covering the entire litter with sterile plastic bags for two hours were placed in zip lock bags on ice using forceps. Thereafter, the fecal samples were mixed with 90 mL of sterilized 0.1% peptone solution and homogenized using a stomacher at 250 rpm for 1 min. More so, the samples were serially diluted ten-fold in peptone solution. A total of 100µL was surface plated on deMan–Rogosa–Sharpe (MRS) (Remel Inc, Lenexa, KS, USA), Brain Heart Infusion (BHI) (BD, Sparks, MD, USA), modified *Bifidobacterium* Iodoacetate Medium-25 (mBIM-25) agar (Himedia, Kennett Square, PA, USA), and MacConkey agar (Remel Inc., Lenexa, KS, USA), to enumerate *Lactobacillus* spp., total bacterial count, *Bifidobacterium*, and *E. coli*, respectively. The plates were incubated for a period of 24 h at 37 °C for the total bacterial count and *E. coli*. Additionally, the MRS and BIM-25 plates were incubated anaerobically for 48–72 h before bacteria counts from all samples were carried out by the plate counting method.

### 2.9. Statistical Analysis

Growth performance, IgY ELISA concentration, differential leukocyte analysis, and DTH data collected were subjected to one-way ANOVA (Statistical Analysis Software (SAS) (2004) Version 9.2. SAS Institute Inc., Cary, NC, USA). On the other hand, fecal microbiota data were subjected to log10 transformation before analysis by one-way ANOVA. All data are presented as the mean ± SEM. Duncan’s multiple-range test was used to determine significant differences among means. Differences were considered statistically significant at *p* < 0.05.

## 3. Results

### 3.1. Growth Performance

From d 1 to d 28, Average Body Weight (ABW), Average Weight Gain (AWG), and Average Feed Intake (AFI) were not significantly different (*p* > 0.05) for all treatments. However, the FCR was significantly influenced by the treatments (*p* < 0.05), with SDP3 having a lower FCR (1.248) value while CX, MX, SDP1, and SDP2 had higher FCR values. Suggesting better feed utilization at the SDP supplementation level of 30 g/kg diet compared to CX and MX (Table 2).

### 3.2. Differential Leukocyte Counts

To investigate the possibility of SDP causing an increase in the percentage of granulocytic leukocytes in the peripheral blood of poultry as well as the percentage of immune cells, we carried out differential leucocyte count analysis. On d 14, the percentage of heterophils for all treatments was significantly different (*p* < 0.05), with SDP supplementation having similar values to MX, while CX had a much higher percentage of heterophils (12.25%). However, there were no significant differences (*p* > 0.05) in the percentage of lymphocytes as well as the ratio of heterophils to lymphocytes for all treatments (Table 3).

### 3.3. Delayed-Type Hypersensitivity (DTH) Reaction

**Figure 1:**
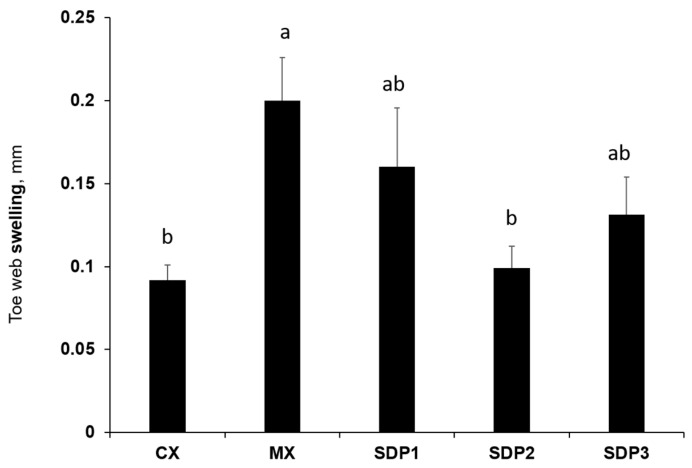
Effect of SDP supplementation on delayed-type hypersensitivity response (d 24). The data are expressed as means ± SEM, with *n* = 8 per treatment; a, b means without a common superscript are different. Statistically significant differences are indicated with *p* < 0.05.

The delayed-type hypersensitivity reaction is an inflammatory response that mainly involves T cells [16]. The DTH response at 24 h post-phytohemagglutinin injection carried out on d 24 showed significant differences (*p* < 0.05) between all treatments, with MX having a higher response compared to CX (Figure 1). The DTH response of SDP treatments (i.e., SDP1, SDP2, and SDP3) was somewhat in between that of CX and MX.

### 3.4. Concentration of Indicator Microorganisms in the Fecal of Broiler Chicks

Total bacteria count was similar for MX and SDP supplemented diet with the exception of SDP2, which had 6.88 Log_10_ CFU/g fecal content. E. coli counts were lower for SDP-supplemented treatments compared with MX, which had a value of 8.86 Log_10_ CFU/g fecal content. In addition, while the population of Lactobacillus was not significantly different (*p* > 0.05), the number of Bifidobacteria was significantly different (*p* < 0.05), with MX and SDP2 and SDP3 having a much higher population compared to SP1 and CX (Table 4).

### 3.5. Plasma Total IgY antibody Concentration

**Figure 2:**
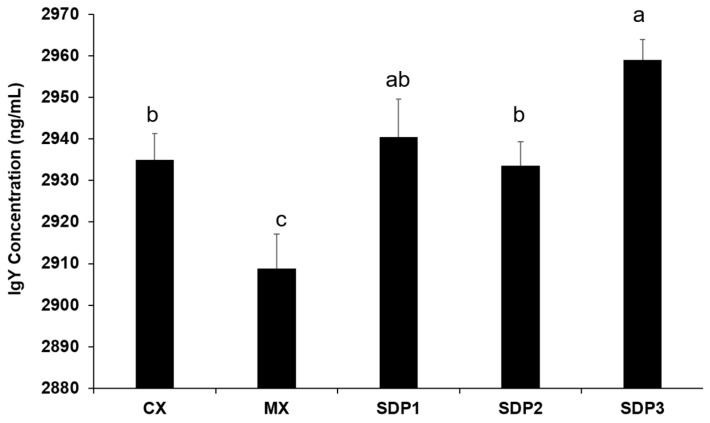
Effect of dietary spray-dried plasma on Serum IgY Concentration (d 25). The data are expressed as means ± SEM, with *n* = 8 per treatment; a, b, c means without a common superscript are different. Statistically significant differences are indicated with *p* < 0.05.

To investigate the amount of circulating IgY antibodies in the plasma of chicken-fed SDP and MX diets, an ELISA assay was performed. Results demonstrated that SDP supplementation significantly increased (*p* < 0.05) the concentration of IgY in circulation compared to MX and was similar to CX (Figure 2).

### 3.6. Pro-Oxidant Capacity

**Figure 3:**
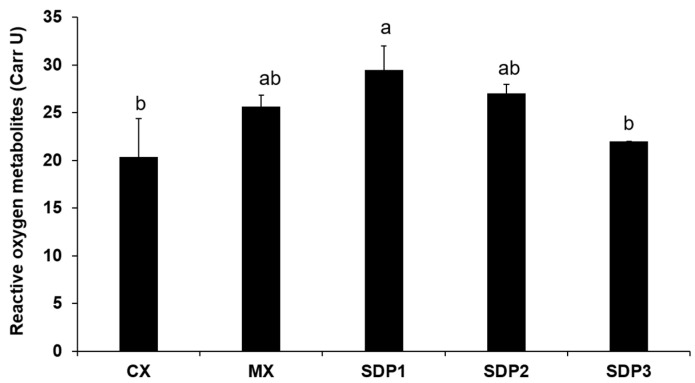
Effect of SDP supplementation of broiler diets on plasma oxidative status. The data are expressed as means ± SEM, with *n* = 8 per treatment; a, b means without a common superscript are different. Statistically significant differences are indicated with *p* < 0.05.

The effect of SDP supplementation on oxidative status in plasma is shown in Figure 3. SDP supplementation significantly influenced (*p* < 0.05) ROMs (reactive oxygen metabolites). SDP1 had the highest value for a pro-oxidant capacity at 29.5 mg Carr U, which is >27.20 Carr U and, therefore, indicates oxidative stress. Similarly, oxidant stress was indicated for MX and SDP2; however, oxidative stress was not observed in birds in the CX and SDP3 groups, where the ROMs were within the normal range (20.3 ± 4.08 and 22 ± 0.00 mg Carr U, respectively.

## 4. Discussion

Spray-dried plasma contains biochemical components that influence metabolic processes as well as immune responses [7]. SDP can also serve as a suitable alternative to antibiotics in broiler chicken feed as it showed similar efficacy in reducing cecal *Salmonella* [13]. In the present study, we evaluated the immunological and biochemical profile of SDP in order to understand how SDP enhances performance values when supplemented to the diet. Our results demonstrated that SDP not only improved the FCR of broiler chickens over a period of 4 weeks, but it also increased the concentration of circulating antibodies and reduced the number of heterophils.

In the present study, supplementing broiler diets with SDP improved the FCR of broiler chickens. However, previous studies using broiler chickens have reported an increase in other performance parameters. For example, Walters et al. [17] reported an increase in body weight at d 25 and d 42. Moreover, in challenge studies, SDP has been reported to reduce mortality and increase body weight during the starter and finisher phases compared to the control group [18]. The disparity in our findings may be a result of the difference in the duration of the experiment, suggesting that the SDP addition to the starter diet may have a long-term benefit on the growth performance of broiler chickens. Studies on SDP supplementation in the diet of piglets have shown that SDP strengthens intestinal barrier function and improves intestinal morphology [19]. While the improvement in growth parameters may be attributed to the improvement in intestinal development, other factors/co-factors may be responsible. In order to further understand how SDP enhances FCR in broiler chickens, we performed biochemical and immunological studies.

The d-ROM test is a standard for measuring pro-oxidant capacity as it measures the blood concentration of hydroperoxides, which belong to the reactive oxygen metabolites group. These hydroperoxides are produced by the oxidation of molecules such as amino acids, glucosides, peptides, lipids, and proteins that are present in spray-dried plasma [7]. Our pro-oxidant test results demonstrate that the level of oxidative stress decreased with increasing SDP supplementation, with SDP3 falling within the normal range similar to CX, while MX, SDP1, and SDP2 showed a low level of oxidative stress. This suggests that an increase in the supplementation of SDP decreased the amount of hydroperoxide in the blood, thereby lowering oxidative stress in the system. The accumulation of free radicals in animal cells leads to oxidative stress, a condition that is detrimental to animal health and may result in reduced performance, disease, and death [20,21]. Interestingly, SDP3 had the best FCR among the treatments. In addition, immunological parameters such as circulating levels of heterophils decreased with SDP supplementation. Heterophils play an important role in innate immunity and resistance to infection [22]. In the present study, the diet supplemented with SDP and MX had a lower percentage of heterophils compared to the control, suggesting that there was no infection or inflammation present [23]. The interaction of cells involved in inflammatory responses and cytokine production at the site of antigen exposure influences delayed-type hypersensitivity (DTH). Therefore, the effective interaction of these cells and other factors required to trigger an immune response in response to stimuli leads to the detection of an intense DTH response [24]. In this study, MX had the most vigorous response and was similar to SP1 and SP3. Thus, our result demonstrated that SDP-supplemented treatments, and, to a larger extent, MX, can trigger an immune response to infection.

In addition, immunoglobulin Y concentrations were higher in the SDP-supplemented treatment compared to MX. These higher IgY concentration levels may indicate the chick’s capacity to develop an efficient immune system sooner. In a study that carried out immunization using chicken immunoglobulin Y (IgY), chicken IgY was highly effective against a variety of intestinal pathogens and enhanced mucosal barrier integrity in different animals [25,26]. The gastrointestinal mucosa secretes immunoglobulins that help in the maintenance of its epithelial barrier by transporting them back into the lumen or by facilitating their removal [27,28]. In addition to neutralizing pathogens at mucosal surfaces, immunoglobulins also function in the regulation of gut microbiota [29]. This was reinforced by our fecal bacteria population result, which showed a statistical increase in the amount of *Bifidobacterium*, a beneficial microorganism of the gastrointestinal tract. *Bifidobacterium* can cause changes to the gut microbiota and modulate T regulatory cell functional metabolism, posing an immune checkpoint blockade to harmful pathogens [30]. This suggests that SDP’s ability to prevent infection and increase the concentration of immunoglobulin without elevating oxidative stress levels possibly plays a key role in supporting intestinal development and gut microbiota modulation.

## 5. Conclusions

In conclusion, our data demonstrated that SDP, and particularly SDP3, could serve as a dietary supplement in lieu of antibiotics in broiler chicken diets as it improved the feed conversion ratio and modulated gut microbiota. Moreover, the use of SDP was shown to be safe and did not cause the buildup of ROS that lead to oxidative stress. Most importantly, we determined that the decrease in heterophils and increase in immunoglobulin concentration supported intestinal barrier integrity and promoted an increase in the population of *Bifidobacterium,* which is beneficial for gut homeostasis. In conclusion, improvement in intestinal development was the key factor in the enhanced growth performance in broiler chickens.

## Figures and Tables

**Table 1:** Composition of experimental diets ^1^ (% “as is”).

Ingredient	Control Diet	BMD Diet ^1^	SDP1 Diet ^1^	SDP2 Diet ^1^	SDP3 Diet ^1^
Corn	50.59	50.59	52.07	53.54	55.02
Soybean meal	40.67	40.67	38.72	36.78	34.83
Spray-dried plasma (SDP, AP920)	0.00	0.00	1.00	2.00	3.00
Poultry fat	4.53	4.53	4.10	3.68	3.26
Limestone	1.37	1.37	1.39	1.42	1.45
Mono-Dicalcium phosphate	1.51	1.51	1.47	1.43	1.39
Salt NaCl	0.24	0.24	0.21	0.19	0.16
Soda bicarbonate	0.16	0.16	0.12	0.08	0.04
L-Lysine HCl 98%	0.17	0.17	0.16	0.14	0.13
DL-Methionine 99.0%	0.34	0.34	0.33	0.33	0.32
L-Threonine 98.5%	0.10	0.10	0.09	0.08	0.07
NCSU Poultry Vitamin Premix ^2^	0.05	0.05	0.05	0.05	0.05
NCSU Poultry Mineral Premix ^3^	0.20	0.20	0.20	0.20	0.20
Bacitracin (Antibiotic, g/kg)	----	0.055	----	----	
Choline chloride 60%	0.07	0.07	0.10	0.10	0.10
Analyzed nutrient composition ^4^					
Metabolizable energy (Kcal/kg)	3146	3181	3150	3111	3150
Crude protein, %	23.71	24.86	23.79	23.96	23.86
Crude fat, %	6.17	6.31	5.84	5.42	5.32
Crude fiber, %	2.3	2.3	2.3	2.2	2.2
Ash, %	5.76	5.85	5.76	5.61	5.49
Calculated nutrient composition					
Total sulfur amino acids, %	1.04	1.04	1.06	1.06	1.06
Lysine, %	1.42	1.42	1.43	1.43	1.43
Calcium, %	0.96	0.96	0.95	0.95	0.95
Available phosphorus, %	0.48	0.48	0.48	0.48	0.48

^1^ Diets used in this study include the following: (i) unmedicated corn–soybean meal (**SBM**) basal without SDP (Control diet); (ii) unmedicated corn–SBM basal into which bacitracin methylene disalicylate (**BMD**) was added at 0.055 g/kg diet (BMD diet); and (iii) SDP1, SDP2, and SDP3 diets in which SDP was added to unmedicated corn–SBM basal at 1% (10 g/kg diet), 2% (20 g/kg diet), and 3% (30 g/kg diet), respectively. ^2^ Vitamin Premix, supplied per kilogram of diet: Vitamin A (6600 IU), Vitamin D (1980 IU), Vitamin E (33 IU), Vitamin B12 (0.02 mg), Biotin (0.13 mg), Menadione (1.98 mg), Thiamine (1.98 mg), Riboflavin (6.60 mg), d-Pantothenic Acid (11.0 mg), Vitamin B6 (3.96 mg), Niacin (55.0 mg), and Folic Acid (1.1 mg). ^3^ Mineral Premix, supplied per kilogram of diet: Manganese (Mn), 60 mg; Zinc (Zn), 60 mg; Iron (Fe), 40 mg; Copper (Cu), 5 mg; Iodine (I), 1.2 mg; and Cobalt (Co), 0.5 mg. ^4^ Experimental diets were analyzed for proximate nutrient composition by Eurofins Scientific Inc. Nutrient Analysis Center, 2200 Rittenhouse Street, Suite 150, Des Moines, IA 50321.

**Table 2:** Effect of SDP supplementation on growth performance of broiler chicks (d 1 to d 28).

Treatment	Average Body Weight (kg/bird) ^1^	Average Weight Gain (kg/bird) ^1^	Average Feed Intake (kg/bird) ^1^	FCR (kg:kg) ^2^
CX	1.58	1.49	1.96	1.318 ^a^
MX	1.47	1.36	1.76	1.294 ^ab^
SDP1	1.47	1.40	1.81	1.292 ^ab^
SDP2	1.44	1.42	1.86	1.311 ^a^
SDP3	1.57	1.52	1.91	1.258 ^b^
SEM	0.051	0.052	0.082	0.015
*p*-value	0.328	0.191	0.160	0.022

^a,b^ Mean values with superscript letters that are different within a column are significantly different (*p* < 0.05). ^1^ Values are based only on the weight of live birds. ^2^ FCR = Feed conversion ratio, calculated as feed-to-gain ratio and adjusted for mortality by including the gains of dead birds in the calculations.

**Table 3:** Effect of SDP supplementation on number of circulating leucocytes (d 14).

Treatment	Heterophils (%)	Lymphocytes (%)	H:L Ratio ^1^
CX	12.25 ^a^	65.50	0.19
MX	4.92 ^c^	63.60	0.09
SDP1	7.90 ^b^	74.35	0.11
SDP2	6.40 ^bc^	73.82	0.10
SDP3	7.62 ^b^	72.50	0.11
SEM	1.849	4.724	0.032
*p*-value	0.049	0.499	0.144

^a–c^ Mean values with superscript letters that are different within a column are significantly different (*p* < 0.05). ^1^ Heterophil to lymphocyte ratio.

**Table 4:** Effect of SDP on bacterial population (Log10 CFU/g) in fecal samples.

Treatment	Total Bacteria Count	*E. coli*	*Lactobacillus* spp.	*Bifidobacterium* spp.
CX	8.11 ± 0.07 ^b^	7.49 ± 0.05 ^c^	9.00 ± 0.18	7.22 ± 0.01 ^b^
MX	8.72 ± 0.16 ^a^	8.86 ± 0.16 ^a^	8.91 ± 0.05	7.98 ± 0.09 ^a^
SDP1	8.40 ± 0.20 ^ab^	7.25 ± 0.07 ^c^	9.07 ± 0.05	7.23 ± 0.05 ^b^
SDP2	6.88 ± 0.05 ^c^	6.49 ± 0.06 ^d^	8.99 ± 0.11	7.70 ± 0.11 ^a^
SDP3	8.79 ± 0.17 ^a^	8.01 ± 0.08 ^b^	8.96 ± 0.13	7.81 ± 0.13 ^a^
*p*-value	0.0001	0.0001	0.906	0.003

^a–d^ Mean values with superscript letters that are different within a column are significantly different (*p* < 0.05).

## Data Availability

Data will be made available on reasonable request.

## Sample Availability

Samples of the compounds are not available from the authors.
